# Effect of Samples Size on the Water Removal and Shrinkage of *Eucalyptus urophylla × E. grandis* Wood during Supercritical CO_2_ Dewatering

**DOI:** 10.3390/ma15228073

**Published:** 2022-11-15

**Authors:** Honghai Liu, Zhilan Li, Xiaokai Zhang, Simin Zhou

**Affiliations:** 1 College of Furnishings and Industrial Design, Nanjing Forestry University, Nanjing 210037, China; 2 Jiangsu Co-Innovation Center of Efficient Processing and Utilization of Forest Resources, Nanjing Forestry University, Nanjing 210037, China

**Keywords:** moisture distribution, moisture transfer, shrinkage, supercritical CO_2_ dewatering, *Eucalyptus urophylla*

## Abstract

*Eucalyptus urophydis E. grandis* green wood with different lengths were dewatered using CO_2_ that was cyclically alternated between the supercritical fluid and gas phases. The results indicate that shorter specimens can be dewatered to below the fiber saturation point (FSP). There was no significant difference in the dewatering rate between the specimens of 20 and 50 mm in length. The dewatering was faster when the moisture content (MC) was over the FSP, leading to a greater gradient and a non-uniform distribution of moisture. The MC distributions in all specimens had no clear differences between in tangential and radial directions. Supercritical CO_2_ dewatering generated a different moisture gradient than conventional kiln drying. Most water was dewatered from the end-grain section of the wood along the fiber direction, but a small amount of water was also removed in the transverse directions. There was no deformation in the specimens when the MC was above the FSP.

## 1. Introduction

Green wood produced from logs usually has a high moisture content (MC). For particular softwood species, the MC can exceed 200% based on the wood material’s oven-dry weight and shows large within-tree variation [1]. Removing water from wood to a reasonable point is a crucial step in wood processing because it influences the wood’s stability [2,3] during its service life and the subsequent modification and treatment with various agents to prolong its life [4,5].

The drying of green wood involves two distinct phases and consists of a complex of physical and mechanical processes. In the first drying phase, liquid free water in the wood cell lumina is removed to a critical point where bound water is mostly retained in the wood cell. This critical point is termed the fiber saturation point (FSP) [6]. The FSP is approximately 30% and mainly varies with species and temperature [7]. The FSP of *Eucalyptus urophydis* is 29%, which was obtained via two classical methods [8]. In the second drying phase, bound water in the cell wall is removed until an equilibrium moisture content (EMC) is reached. This period requires much more energy compared with the first drying phase. The removal of water results in increased drying stress, which leads to wood distortion, collapse, and checking.

A suitable choice for the drying method is usually based on a consideration of the drying quality, drying cost, drying time, wood species, and product requirement. Removing water from wood can be achieved with the application of heat to wood using several methods, such as conventional kiln drying, superheated steam kiln drying, vacuum drying, microwave or radio frequency drying, and solar drying [9,10,11,12,13]. All these methods require the phase change of water from liquid to vapor. Water in wood can also be removed by applying mechanical rather than thermal processes. These mechanical dewatering approaches include compression rolling [14], centrifugation [15,16], and pressure expulsion [17,18].

Among them, high-pressure supercritical CO_2_ (ScCO_2_) dewatering has recently been attracting attention. This approach cycles a CO_2_ fluid between the supercritical and gas phases in a system. A large change in CO_2_ volume occurs as its phase changes from supercritical fluid to gas when the pressure is decreased to below the critical point. This results in an efficient fast mechanical expulsion of free water in the cell lumina [17,19,20,21,22,23]. ScCO_2_ dewatering is not only a rapid water removal technique but can also reduce wood deformation [20,24,25,26]. The materials used for ScCO_2_ dewatering tests involved hardwood timber species and softwood species [26]. Meanwhile, the advanced technology of magnetic resonance imaging (MRI) and nuclear magnetic resonance (NMR) spectroscopy [22,27,28,29,30,31,32] were applied to the studies to obtain a deeper insight into the mechanism of the supercritical CO_2_ dewatering process. These studies showed that CO_2_ first entered the latewood and then diffused into the adjacent early wood, while less CO_2_ entered the earlywood directly. CO_2_ fills voids as supercritical fluid and becomes dissolved in water in the lumina of cells, gradually diffusing deeper into the wood. Releasing the pressure lowers the solubility of CO_2_ in water, generating bubbles of CO_2_ that expel water from the cell lumina. Models of the dewatering process have also been developed to deeply understand the dewatering mechanism using mathematical tools to treat the mass transfer and diffusion of supercritical carbon dioxide [33,34], and they obtained good agreement with the experimental results. Most studies concluded that fresh wood could be maximally dewatered to approximately 40% MC [17,20,26,35], but Aggarwal et al. [19] dewatered pine chips to below 15% MC in an ScCO_2_ dewatering experiment.

Experiments on wood ScCO_2_ dewatering have mainly been carried out in laboratories using short samples [17,20,36]. The lengths of the samples used in previous studies were between 95 and 200 mm, and the dimensions of the transverse sections (tangential × radial) ranged from 8 × 8 mm to 37 × 37 mm. Some studies investigated the mechanism of CO_2_ migration and water transfer using MRI and NMR technology but focused on the characteristics of water transfer in wood transverse sections. Only Dawson et al. [20] compared the effect of wood cross-sectional dimensions on the dewatering, but there has been no investigation on the effect of wood length on ScCO_2_ dewatering.

In the present study, *Eucalyptus urophydis E. grandis* specimens with cross-sectional dimensions of 30 × 30 mm and longitudinal dimensions of 2, 5, 20, and 50 mm were dewatered using a supercritical CO_2_ fluid at a pressure of 20 MPa and a temperature of 35 °C. The objectives were to investigate whether short wood can be dewatered from a green MC to an MC below the FSP and the effect of wood length on the characteristics of moisture transfer.

## 2. Materials and Methods

### 2.1. Materials

**Figure 1:**
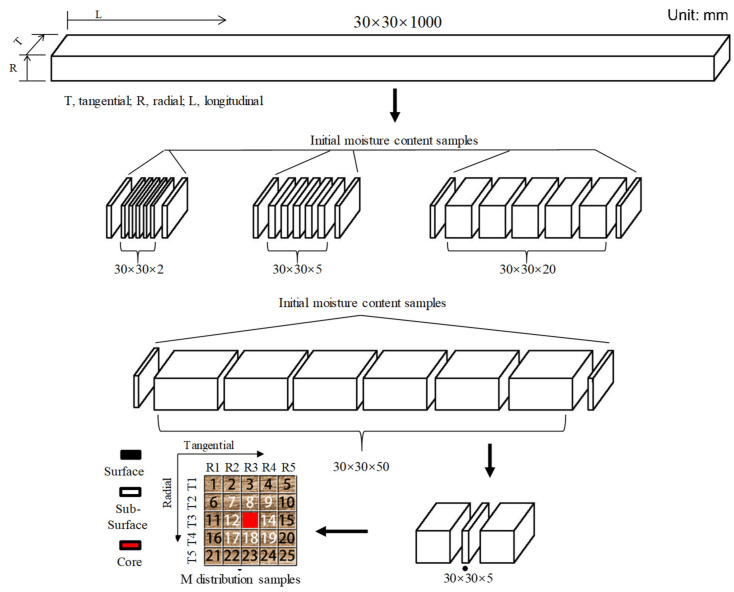
Wood sampling diagram.

One *Eucalyptus urophydis E. grandis* plantation tree was obtained from Guangxi province, China. After felling, 1000 mm long log sections were cut from the tree, end-sealed with wax, wrapped tightly with a plastic film, and then transported to the laboratory of Nanjing Forestry University. Thereafter, the log sections were processed into square sawn heartwood timbers with specifications of 30 (*tangential*) × 30 (*radial*) × 1000 (*longitudinal*) mm in the laboratory. The square sawn timber was double-plastic-bagged and refrigerated at 4 °C until it was treated. One 1000 mm long square timber was selected and then sawn into four end-matched sets of specimens with lengths of 2, 5, 20, and 50 mm (Figure 1). This resulted in a total of five replicates for each specimen size. The initial MC of the specimens was approx. 95%. All specimens were marked and measured immediately. Then, they were put into the pressure vessel for the subsequent dewatering test.

### 2.2. Equipment

**Figure 2:**
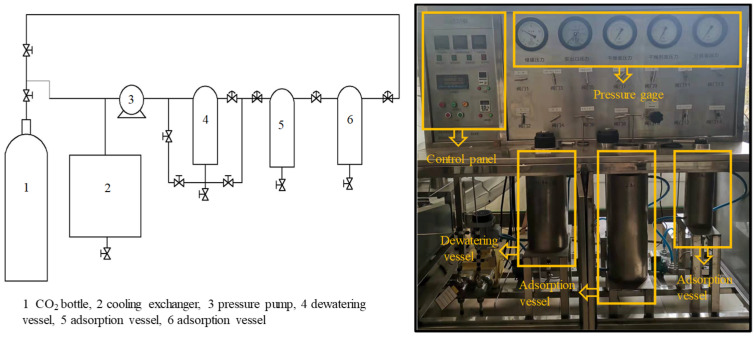
Configuration and photos of the supercritical CO_2_ dewatering plant.

The apparatus used for dewatering was a ScCO_2_ dewatering plant (HM120-50-025, Haian Hongmai Machinery Co., Ltd., Nantong, China). As shown in Figure 2, the plant consisted of a CO_2_ storage bottle (1), a cooling exchanger (2), a pressure pump (3), a 5 L (ϕ100) dewatering vessel (4), and two adsorption vessels. The pressure and temperature of the dewatering vessel could be controlled from atmospheric (0.1 MPa) to 30 MPa and from 30 to 70 °C, respectively. Liquid CO_2_ was supplied to the plant from the storage bottle, and the supercritical pressure was achieved by an air-operated pressure pump. The temperature of the drying vessel was regulated by a heated mantle during the dewatering process.

Other apparatuses included an electric heating oven (DHG-905386-Ⅲ, Shanghai Cimo Medical Instrument Co., Ltd., Shanghai, China), an electronic balance with an accuracy of 0.001 g (HC2004, Huachao high tech equipment Co., Ltd., Shanghai, China), and an electronic vernier caliper with an accuracy of 0.01 mm (CD-20CPX, Mitutoyo Corporation, Tokyo, Japan).

### 2.3. Dewatering Tests

All specimens were inserted into the pressure vessel (Figure 2) after the measurement of the weight and dimensions. Then, the dewatering process started in the plant according to the parameters shown in Table 1. Liquid CO_2_ was used to generate ScCO_2_. The liquid CO_2_ was pumped using a pump that delivered the CO_2_ from the storage bottle into the dewatering vessel at a target pressure. All specimens were held in contact with ScCO_2_ at 20 MPa and 35 °C for 15 min. Then, the pressure was decreased to atmospheric pressure (0.1 MPa) in 10 min by releasing CO_2_ gas through a valve. All specimens were removed from the dewatering vessel and divided into four sealable bags by size for a further 50 min. After CO_2_ gas emission at room temperature, they were weighed and measured again. This completed one full cycle. All specimens were returned to the dewatering vessel for the next dewatering cycle. In total, twelve cycles were repeated in this study.

### 2.4. Moisture Content Determination and Its Distribution

After 12 cycles of dewatering, three specimens of each size were selected for the determination of the MC distribution. The 2, 5, and 20 mm specimens were carefully marked and dissected directly into 25 blocks (Figure 1); however, for the 50 mm specimens, a 5 mm thick slice was sawn in the middle of the selected specimens, which were then marked and dissected. After dissection, wood blocks were promptly weighed to minimize the mass loss due to evaporation. Subsequently, all blocks were dried at 103 °C until a constant oven-dry weight was obtained. The MC was determined using Equation (1) based on the oven-dry weight of each wood block according to the national standard of GB/T 1931-2009:

(1) $$MC=\frac{m_{1}-m_{0}}{m_{0}}$$

where *MC* is the moisture content of a specimen, *m*_1_ is the initial mass of the specimen, and *m*_0_ is the oven-dry mass of the specimen.

### 2.5. Wood Deformation Measurement

The dimensions of a specimen in the tangential and radial directions (Figure 1) were measured using an electronic vernier caliper (CD-20CPX, Mitutoyo Corporation Mitsuyo, Tokyo, Japan) prior to the dewatering experiment and again after each dewatering cycle. The deformation of the wood is presented as shrinkage and was calculated using Equation (2):

(2) $$S=\frac{L_{i}-L_{f}}{L_{i}}$$

where *S* is shrinkage in the tangential or radial directions of a specimen after dewatering, *L_i_* is the initial dimension of the specimen before the dewatering experiment, and *L_f_* is the dimension of the specimen after dewatering.

## 3. Results and Discussion

### 3.1. Characteristics of Dewatering Curves and Dewatering Rate

**Figure 3:**
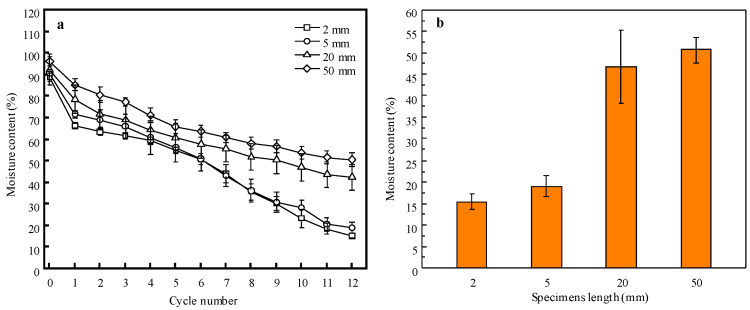
(**a**) Dewatering curves of *Eucalyptus urophylla* specimens with a hold time of 15 min, a temperature of 35 °C, and a pressure of 20 MPa. (**b**) Final average MCs of specimens after 12 dewatering cycles. The error bars in are the mean square errors of the five specimens, indicating their MC deviation after each cycle of dewatering.

In the supercritical CO_2_ dewatering process, CO_2_ penetrates and fills voids as a supercritical fluid and dissolves in water. When the pressure is released, CO_2_ bubbles are generated due to the lowered solubility of CO_2_ in water, which expels water from the cell lumina. As a consequence, the expelling path affects the moisture flow resulting from the supercritical CO_2_ dewatering. Figure 3a,b show the MCs of the specimens after each cycle of dewatering and the final MC after the 12th cycle of dewatering, respectively. For all specimens, a dramatic MC reduction was found after the first dewatering, indicating that a great amount of water was removed in this cycle. In the subsequent dewatering, the MCs of the 20 and 50 mm specimens decreased gradually and showed similar tendencies until the end. The MCs of the 2 and 5 mm specimens also exhibited slow declines in the early cycles, but greater reduction occurred from the 7th cycle compared with the 20 and 50 mm specimens. Their final MCs after the twelfth cycle were 15.3 and 18.9%, respectively (Table 2), which were much lower than the FSP obtained by Yang et al. [8] and the previous results [17,20,26,35]. This indicates the ScCO_2_ can dewater wood to below the FSP. The lower MC after dewatering in this study can be attributed to the short lengths of the specimens, which resulted in the easier penetration of supercritical carbon dioxide into the wood and the expulsion of water. The error bars of the 20 mm specimens are larger than the others; this is attributed to the larger size of the specimens when the MC was determined. The length was 20 mm, while the lengths of the other specimens were less than 5 mm.

Table 2 compares the initial and final MCs, the dewatering times, and dewatering rates of all specimens when the MCs were above and below the FSP. It can clearly be seen that the specimens of 2 and 5 mm in length had greater dewatering rates compared with the 20 and 50 mm specimens. The dewatering rates of the 2 and 5 mm specimens above the FSP were 1.3 and 2.0 times greater than those below the FSP, respectively, indicating faster dewatering when the wood had free water. This indicates that the removal of free water in the cell lumina is easier than the bound water in the cell walls. Comparing the dewatering rates of the 2 and 5 mm specimens, they were quiet different when the MCs were above and below the FSP. However, with a larger length, the dewatering of the 5 mm specimens was faster than that of the 2 mm specimens when the MC was above the FSP. This could be attributed to the higher initial MC of the 5 mm specimens. This also indicates that the length has little effect on free water removal for short specimens. However, an opposite behavior for dewatering was found when the MC was below the FSP, i.e., the dewatering rate of the 5 mm specimens was lower than that of the 2 mm specimens. The free water fraction was mainly displaced by ScCO_2_, but the bound water was removed by diffusion [19]. The larger length of the specimens slowed the penetration of ScCO_2_ into the specimens and the bound water diffusion during the bound water dewatering.

### 3.2. Characteristics of Moisture Distribution and Moisture Gradient

**Figure 4:**
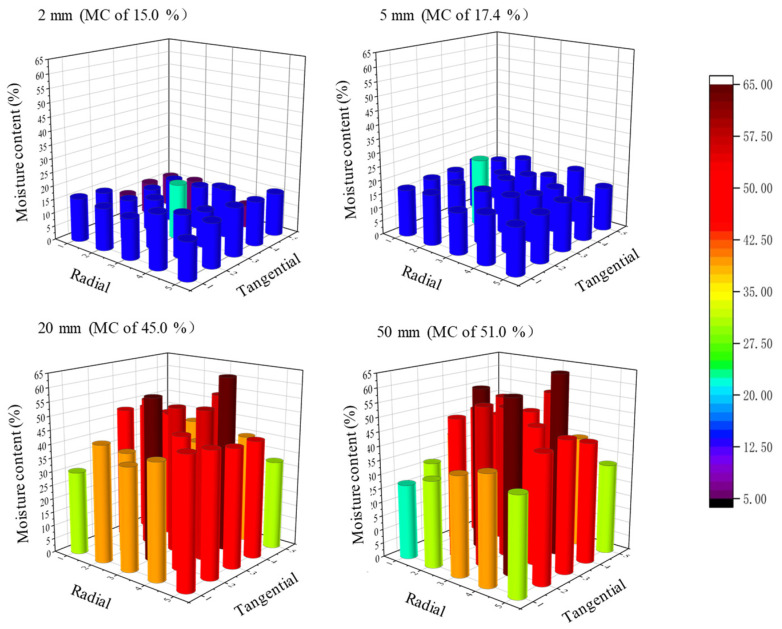
Three-dimensional moisture distributions of specimens with lengths of 2, 5, 20, and 50 mm after the 12th dewatering cycles.

**Figure 5:**
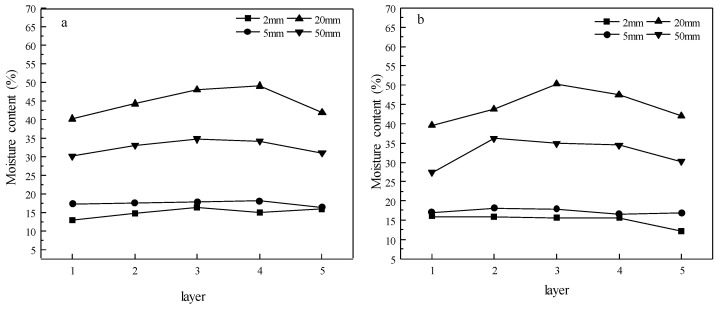
Moisture content distributions in the (**a**) tangential and (**b**) radial directions of specimens with lengths of 2, 5, 20, and 50 mm after the 12th dewatering cycles.

Figure 4 shows a three-dimensional moisture distribution of specimens with lengths of 2, 5, 20, and 50 mm after the 12th dewatering cycles. Figure 5a,b show the tangential and radial moisture distribution of the specimens, respectively. The moisture distribution was almost uniform for the 2 and 5 mm specimens when their MCs were lower, but an extremely non-uniform distribution was observed for the 20 and 50 mm specimens, as they had higher MCs. This coincides with the results of a previous study [17]. The tangential and radial moisture distributions (Figure 5a,b) were uniform at lower MCs, but they became clearly uneven at higher MCs, showing lower MCs in both the tangential and radial surface layers and a higher MC in the core. There were no significant differences in the radial and tangential moisture gradients for all specimens with both lower and higher MCs. The moisture after ScCO_2_ dewatering showed a similar distribution mode, namely the surface MC was much lower than that in the subsurface layers and core [37].

**Figure 6:**
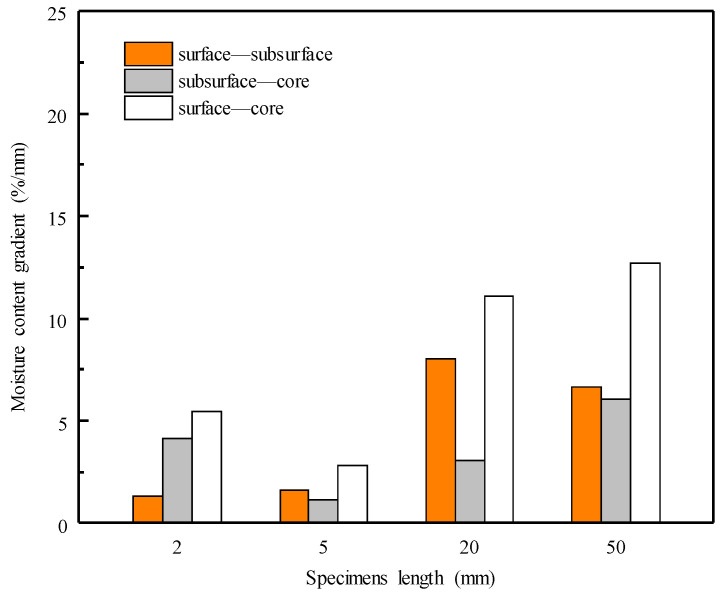
Moisture content gradients in the cross sections of specimens with lengths of 2, 5, 20, and 50 mm after the 12th dewatering cycles.

Figure 6 shows the moisture gradients of specimens with lengths of 2, 5, 20, and 50 mm after the 12th dewatering cycles. The moisture gradient between the surface and subsurface was determined by the difference in the average MCs in the subsurface layers and surface layers (Figure 1). Similar determinations were applied to the subsurface–core and surface–core gradients. The moisture gradients of the 2 and 5 mm specimens were clearly smaller than those of the 20 and 50 mm specimens, coinciding with the moisture distributions in Figure 4 and Figure 5. This indicates that non-uniform moisture distribution results in a greater moisture gradient. Moreover, the moisture gradients of the 5, 20, and 50 mm specimens increased with specimens size, exhibiting almost the same tendency, namely that the greatest gradient was between the surface and core layers, and the smallest gradient was between subsurface and core layers. The water removal in the surface layers was faster than that in the core, leading to an increase in the moisture gradient. However, the moisture gradient between layers was different from that in conventional kiln drying [13], which has the greatest moisture gradient between the surface and subsurface layers. This could be attributed to the special water removal mechanism of ScCO_2_ dewatering, namely the mechanical expulsion of free water in the cell lumina.

### 3.3. Characteristics of Moisture Flow

Using the dimensions of the samples, the surface areas and the ratios of the side surface areas to the total when the MCs were above and below the FSP were calculated. When the MCs were above the FSP, the side surface areas of the 5 and 50 mm specimens were 2.5 times those of the 2 and 20 mm specimens, respectively; however, the dewatering rates of the 5 and 50 mm specimens were 1.15 and 0.98 times those of the 2 and 20 mm specimens, respectively (Table 2). These findings indicate that free water is mostly removed through end surfaces. The penetration of ScCO_2_ into specimens is easier in the wood length direction, resulting in a higher dewatering rate during pressure decompression. Dawson et al. [20] also concluded that the specimens with the smallest cross-sectional dimensions dewatered more slowly than the larger specimens. This result further demonstrated that free water is mostly removed in the wood fiber direction, which was consistent with our result. Water removal in the fiber direction became larger when the MC was below the FSP because the dewatering rate of the 2 mm specimens with less side surface area was obviously higher than that of the 5 mm specimens with more side surface area (Table 2). Bound water is removed by diffusion during wood ScCO_2_ dewatering [19]. Water diffuses mostly in the paths with less resistance. This can explain the faster dewatering rate in the wood fiber direction when the MC was below the FSP because the short specimens had the least resistance in the fiber direction. Additionally, the non-uninform moisture distributions in the cross-section (Figure 4) of the wood can also verify that a certain amount of water in the surface layers of the specimens was removed along the tangential and radial directions, resulting in lower MCs in the surfaces layers.

### 3.4. Characteristics of Deformation of Wood

**Figure 7:**
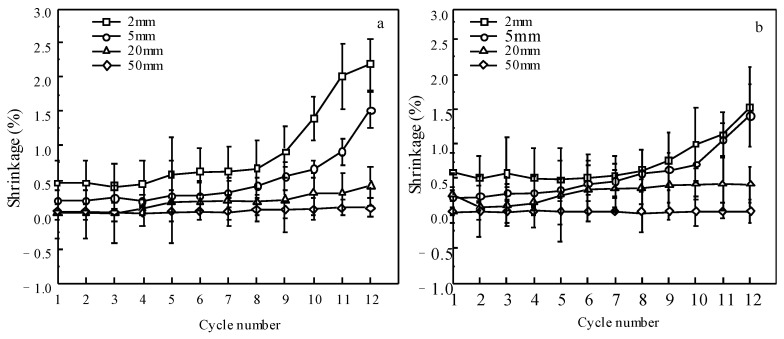
Shrinkage of specimens with lengths of 2, 5, 20, and 50 mm in the (**a**) tangential and (**b**) radial directions.

The shrinkage of specimens in the tangential and radial directions after each cycle is illustrated in Figure 7a,b. For 50 mm specimens, there was no shrinkage after each cycle of dewatering, coinciding with previous findings [17]. However, small shrinkage (0.4%) in the 20 mm specimens, as shown in Figure 7, was observed after the 12th dewatering cycle. This could be attributed to the fact that the MC in the surface layers was lower than the FSP (Figure 5a,b). Wood has the natural property that it shrinks below the FSP due to moisture loss [7]. For the 2 and 5 mm specimens, apparent shrinkages occurred after the 9th dewatering (Figure 7a,b), where the MC of the wood was lower than the FSP (Figure 3a). The shrinkage in the tangential direction was larger than that in the radial direction, showing a tendency similar to conventional kiln drying. This result indicates that wood shrinks, even after the supercritical CO_2_ dewatering process. *Eucalyptus urophydis* wood is a collapse-prone material. Special deformation (collapse) occurs during conventional kiln drying when its MC is above the FSP. The smallest collapse of *Eucalyptus urophydis* wood (30 × 30 × 30 mm) in the mild conventional kiln drying at 25 °C and 80% was about 4% when its MC was 45% [8]. However, there was small deformation in the 20 and 50 mm specimens until their MCs approached approx. 50%. This demonstrates that supercritical CO_2_ dewatering is an effective dewatering technique for preventing the collapse of *Eucalyptus* wood.

## 4. Conclusions

*Eucalyptus urophydis E. grandis* green specimens with lengths of 2, 5, 20, and 50 mm were dewatered using supercritical CO_2_ (ScCO_2_). The dewatering rates of the 2 and 5 mm specimens were larger compared with the 20 and 50 mm specimens. The short specimens could be dewatered below the fiber saturation point (FSP). The dewatering rates of the 20 and 50 mm specimens were not significantly different from each other. The dewatering was faster when the MC was over the FSP, which created a greater MC gradient and an obvious non-uniform distribution. It slowed when the MC was below the FSP, which resulted in a small moisture gradient and a uniform moisture distribution. The MC distributions of all specimens presented no clear differences between the tangential and radial directions. Supercritical CO_2_ dewatering generated a different moisture gradient than conventional drying. Most water was dewatered from the end-grain section of the wood along the fiber direction, but a small amount of water was also removed in the transverse directions. No deformations were observed in the specimens when the MC is above the FSP, indicating that ScCO_2_ dewatering is an effective method for reducing wood collapse.

## Figures and Tables

**Table 1:** Process parameters for ScCO_2_ dewatering.

Process Parameter	Value
Supercritical temperature (°C)	35
Maximum pressure (MPa)	20
Minimum pressure (MPa)	0.1
Pressurization time (min)	20
Hold time (min)	15
Decompression time (min)	10
CO_2_ emission time (min)	50

**Table 2:** The initial and final MCs, dewatering times, and dewatering rates of specimens with lengths of 2, 5, 20, and 50 mm.

Length of Specimens (mm)	MC (%)		Dewatering Time (h)			Dewatering Rate (%/h)		
	Initial	Final	≥FSP	≤FSP	Total	≥FSP	≤FSP	Total
2	82.9	15.3	2.25	0.75	3.0	23.9	18.3	22.5
5	90.9	18.9	2.25	0.75	3.0	27.5	13.5	24.0
20	92.4	46.8	3.0	-	3.0	15.2	-	15.2
50	95.3	50.6	3.0	-	3.0	14.9	-	14.9

Note: the FSP is 29% [8].

## Data Availability

Not applicable.
